# Awareness and acceptability of human papillomavirus vaccination among parents in Tshwane, South Africa

**DOI:** 10.4102/hsag.v30i0.3078

**Published:** 2025-09-30

**Authors:** Mary N. Mlangeni, Ntlogeleng M. Mogale, Mmampedi C. Mathibe, Feni M. Motshwane, Thembelihle S. Ntuli

**Affiliations:** 1 Department of Public Health, School of Health Care Sciences, Sefako Makgatho Health Sciences University, Tshwane, South Africa; 2 Department of Health, Faculty of Child Health Directorate, Integrated School Health Programme, Tshwane, South Africa; 3 Department of Statistical Science, School of Science and Technology, Sefako Makgatho Health Sciences University, Tshwane, South Africa

**Keywords:** human papillomavirus, school-based vaccination programme, parents, girls, acceptability, awareness, knowledge

## Abstract

**Background:**

The human papillomavirus (HPV) vaccination programme is the most effective strategy to reduce the burden of cervical cancer. However, little is known about the parents’ knowledge and awareness of the programme, as they are important role players in its successful implementation.

**Aim:**

To determine the awareness, knowledge and factors associated with HPV vaccination programme acceptability among parents of Grade 5 girls attending public primary schools.

**Setting:**

The study was conducted in Sub-District 6, Tshwane Metropolitan Municipality in the Gauteng province, South Africa.

**Methods:**

A descriptive, cross-sectional survey was conducted among 421 participants from 21 schools utilising a self-administered questionnaire. Frequency distribution and logistic regression were used to analyse data. STATA 17 SE software was used.

**Results:**

About 321 questionnaires (*N* = 321) were returned, constituting a response rate of 76%. The levels of awareness (*n* = 279, 87%) and acceptability (*n* = 286, 89%) were high, with relatively lower, but still significant knowledge (*n* = 215, 67%). Black parents who were aware and knowledgeable about the programme were significantly more likely to accept the vaccination programme.

**Conclusion:**

Despite high levels of programme awareness and acceptability among parents of eligible girls, there remained crucial knowledge gaps regarding programme specifics. Inadequate knowledge may contribute to parents’ low submission of consent forms, resulting in sub-optimal vaccine uptake.

**Contribution:**

Enhanced parental education on HPV vaccination and its proven effectiveness is essential to ensure optimal vaccine uptake, prevent infection and address vaccine hesitancy.

## Introduction

Cervical cancer is preventable through vaccination of young girls with the human papillomavirus (HPV) vaccine, before sexual debut and routine screening for women over the age of 30 years (WHO 2020). Despite these interventions, cervical cancer remains a leading cause of morbidity and mortality in South Africa (Sung et al. 2021), accounting for over 10 000 new cases and 5000 deaths recorded annually among women (GLOBOCAN 2023). Globally, cervical cancer is the fourth most common cancer in women after breast cancer, responsible for approximately 342 000 deaths in 2020, with 90% of these deaths occurring in low- and middle-income countries (Arbyn et al. 2021; WHO 2023). Persistent infection with high-risk HPV subtypes, particularly types 16 and 18, is the primary cause of cervical cancer, contributing to over 70% of cases (De Sanjosé et al. 2021). While HPV vaccination and early detection programmes exist, cervical cancer continues to pose a significant public health challenge, particularly in sub-Saharan Africa, where South Africa carries one of the highest burdens (Okunade et al. 2023).

In 2014, South Africa introduced a school-based HPV vaccination programme under the Integrated School Health Programme (ISHP), initially targeting Grade 4 girls aged 9 years and older in public primary schools (Maseko, Chirwa & Muula 2020). In 2020, the National Department of Health (NDoH) revised the target group to Grade 5 learners, administering a two-dose bivalent HPV vaccine (Cervarix) to protect against HPV-16 and HPV-18 (NDoH 2022). The programme aimed to achieve 80% annual vaccination coverage; but in 2022, data showed only ~65% of eligible girls were vaccinated because of consent form challenges and logistical barriers (Tshivhase, Maseko & Gaffoor 2023). Despite the provision of free and accessible vaccines, these systemic issues persist, underscoring the need for improved community engagement and streamlined consent procedures.

Parental involvement in the successful implementation of the school-based HPV vaccination programme is vital (Jensen, Johansen & Damsgaard 2022). A key challenge is low consent form return rates, often because of:

- Limited parental awareness of HPV and its vaccine (Ndou, Van Zyl & Mashamba-Thompson 2021).
- Misinformation and vaccine hesitancy (Wiyeh et al. 2023).
- Logistical issues (e.g. forms not reaching parents) (Maseko et al. 2020). Studies confirmed that parental knowledge directly influenced vaccine uptake – when parents understand HPV risks, acceptability increases by 50% – 70% (Kisaakye et al. 2022). However, awareness remains low in South Africa, necessitating better community engagement (Tshivhase et al. 2023).

Therefore, this study aims to determine the level of awareness, knowledge and factors associated with the acceptability of the HPV vaccination programme among parents of Grade 5 girls attending public primary schools in the Tshwane Metropolitan.

## Research methods and design

### Study design

This study is a cross-sectional descriptive survey where a self-administered questionnaire was used to collect data from parents of Grade 5 girls in public primary schools.

### Study setting

The study took place in the Tshwane Metropolitan in Gauteng province, South Africa. Tshwane Metro is one of the three metros in Gauteng province after Johannesburg and Ekurhuleni metros. There are 367 public primary schools with about 27 150 girls who are eligible for HPV vaccination (Gauteng Provincial School Enrolment Data 2022). The study was conducted in Sub-district 6, which is 13 km from the Central Business Centre. The area is in the eastern part of the city, and it includes townships and suburban areas such as Mamelodi, Silverton, Eersterust, Nellmapius, Constantia Park and Elarduspark. The study area comprises 60 public primary schools classified into quintiles 1 to 5. Quintile 1 schools are considered the least resourced schools compared to quintile 5 schools (as shown in Table 1).

**TABLE 1:** Classification of schools per strata and estimated sample size.

Quintile	Total number of schools	Proportion (%)	Selection of schools based on weighed proportions plus 20% buffer	Estimated target population	Proportion	Estimated sample size	Required participants per school
1	15	25	5	1396	1396/3936 = 35.4%	421 × 35.4% = 149	30
3	15	25	5	559	559/3935 = 14.2%	421 × 14.2% = 60	12
4	21	35	8	1235	1235/3935 = 31.3%	421 × 31.3% = 132	17
5	9	15	3	745	745/3935 = 19%	421 × 19% = 80	27
**Total**	**60**	**100**	**21**	**3935**	**100%**	**421**	**-**

Note: 20% Buffer (4) added to total schools.

### Study population

The study population comprised approximately 3935 parents or caregivers of Grade 5 girls aged 9 years and above in the public primary schools in the sub-district. The parents were over 18 years old and responsible for the care of the eligible girls attending the primary schools in the area.

### Sampling strategy and sample size determination

A stratified random sampling technique was employed in the study to ensure proportional representation across school quintiles. Schools were first grouped using quintiles (strata), and sampling weights were calculated according to the number of schools in each tier, against the total number of schools. This was used to determine the required number of schools per quintile. Within each quintile, schools were then randomly selected. For sample size determination, Cochran’s formula for proportions was applied based on 95% confidence interval, a 5% margin of error, a 50% distribution rate and an estimated target population of 3935. This yielded a minimum sample size of 351 participants and was subsequently increased by 20% to account for potential non-response bias, resulting in a final sample size of 421. Sampling weights based on the estimated population were calculated and utilised to determine the number of participants in each quintile. Parent selection followed consecutive sampling techniques, with all consenting caregivers included until reaching the predetermined quota for each stratum and school.

### Data collection tool and procedure

The 35-item questionnaire had four main sections with questions on socio-demographic characteristics of parents or caregivers, awareness about HPV and the vaccination programme, knowledge about the vaccination programme and, lastly, acceptability of HPV vaccination among parents or caregivers. The tool was developed in English and translated into three other languages that are commonly used in the area (Northern Sotho, IsiZulu and Afrikaans). To ensure consistency in meaning, two independent translators conducted forward translation, converting the questionnaire from the source language into three additional languages. Their translations were analysed, and inconsistencies were rectified to produce a unified version. A distinct translator was employed to back-translate the questionnaires into the original language to identify any discrepancies from the original meaning. Piloting of the questionnaire was conducted on a small group from the desired sample; the language was altered according to their comments, and then final proofreading was done to ensure uniformity in terminology and formatting.

*Socio-demographic questions* were mainly closed-ended with pre-determined options. Awareness of HPV and the vaccination programme was assessed as ‘Yes’ or ‘No’. Multiple-choice questions were used to assess the level of knowledge on HPV and the vaccination programme. Questions on the acceptability of HPV vaccination were measured using a 5-point Likert Scale ranging from ‘Strongly Disagree’ coded as 0 to ‘Strongly Agree’ coded as 5. The total of 10 questions was calculated and converted into percentages. Scores below 40% were categorised as ‘Not accepting the HPV vaccine’ and coded as zero, while scores above 40% were categorised as ‘Accepting the vaccine’ and subsequently coded as one. This variable served as the study’s dependent variable.

Questionnaires (*N* = 421) were distributed to eligible parents between May and July 2022. Participant packs containing an information leaflet, consent form and the questionnaire were issued by the researcher to select Grade 5 girls in each class at different schools to take home. The questionnaires were collected a week following distribution at each school, after a telephonic reminder and confirmation with the school.

### Data handling and analysis

Google Forms (Google LLC) was used to capture data from the paper-based questionnaires. The responses were automatically populated on the online Google Sheets (Google, LLC). Data were downloaded and imported into Stata 17 SE (Stata Corp, Texas, US) for cleaning, coding and analysis. Descriptive statistics was used to analyse the data. Frequency distribution was used to analyse categorical variables. The results are presented as percentages in tables and graphs. Summary statistics were used to analyse continuous variables to determine measures of central tendency and dispersion, and were presented as means and standard deviations. Logistic regression analysis was used to determine factors associated with the acceptability of HPV vaccination among parents. The logistic regression models were developed using a two-stage variable selection process. First of all, purposive covariate selection was applied based on theoretical relevance, and backward elimination was implemented where all variables showing univariate association at *p* < 0.25 were retained for multivariable analysis (Kleinbaum & Klein 2021). This relaxed threshold helps preserve potentially important confounders during preliminary screening. Another model using the backward elimination process was built. A final model with a smaller log-likelihood ratio, higher pseudo *R*^2^ value, better Hosmer–Lemeshow Goodness of fit test was selected. A *p*-value of < 0.05 in the final multivariable model was considered statistically significant.

### Reliability, validity and bias

The study material sent to participants was translated and administered in four main languages used in the area (English, Afrikaans, IsiZulu and Sesotho). The tool was standardised with the same questions for all participants and clear instructions that guided them on what was expected. Questions asked on the data collection tool answered what were supposed to be addressed (i.e. awareness, knowledge and acceptability of the school-based HPV vaccination programme among parents). Cronbach’s alpha for the acceptability questions was determined, and a scale reliability coefficient of 0.87 was obtained. Before administration, the tool was pilot-tested among 10% of the sample, and the pilot test results were not included in the study. Consecutive sampling was used to select participants, and all eligible parents who expressed willingness to participate were included in the study. The questions were carefully crafted to be as clear as possible, free from jargon and leading questions. The questionnaire was anonymous, and participants were assured of confidentiality, which reduces non-response bias. All data that were supposed to be collected for the study were collected and published.

### Ethical considerations

The study was approved by Sefako Makgatho Health Sciences University Research Ethics Committee (SMUREC) with reference number SMUREC/H/350/2021: PG. Permission to conduct the study at the schools was provided by the Gauteng Department of Basic Education (Ref: 8/4/4/1/2) and Tshwane South Educational District. Additional authorisation was secured from the school principals and governing bodies.

To ensure compliance with government measures and safety considerations during the coronavirus disease 2019 (COVID-19) era, preventative measures such as frequent washing of hands, social distancing, sanitising and wearing of masks were always adhered to during the study. All parents who accepted to take part in the study were asked to sign a written informed consent. Confidentiality of data was maintained by using anonymous questionnaires that were filled in at home, without any personal identifiers. Information collected from participants was not shared with anyone; it was safely stored electronically and solemnly used only for research purposes.

## Results

Out of the 421 distributed questionnaires, 321 were returned, constituting a response rate of 76%. The results show that about 94% (*n* = 301) of the participants were females (Table 2). Most participants were South African (96%, *n* = 307), followed by Zimbabweans (2%, *n* = 6). There were more black parents or caregivers, and 42% were between the ages of 30–39 years, single (68%, *n* = 218), Christians (94%, *n* = 300), with at least a high school education (28%, *n* = 89), with most of them unemployed (55%, *n* = 177).

**TABLE 2:** Socio-demographic characteristics of parents and caregivers of girls eligible to receive human papillomavirus vaccine in Tshwane primary schools.

Variables	Frequency (*n*)	%
**Gender**		
Female	301	93.77
Male	20	6.23
**Nationality**		
Malawian	1	0.31
Swati	1	0.31
Mozambican	3	0.93
Lesotho	3	0.93
Zimbabwean	6	1.87
South African	307	95.64
**Race**		
White parents and caregivers	15	4.67
Parents and caregivers of mixed race	19	5.92
Black parents and caregivers	287	89.41
**Age category**		
< 30 years	78	24.30
30–39 years	135	42.06
40–49 years	90	28.04
50+ years	18	5.61
**Marital status**		
Single	218	67.91
Married	92	28.66
Divorced	7	2.18
Widowed	4	1.25
**Religion**		
None	17	5.31
Christian	300	93.75
Jewish	1	0.31
Muslim	2	0.63
**Highest qualification**		
Primary school level	62	19.31
High school level	89	27.73
Matric level	100	31.15
Tertiary level	70	21.81
**Employment status**		
Unemployed	177	55.14
Self-employed	26	8.10
Casual employee	23	7.17
Employed	95	29.60
**HPV (source of information)**		
Never heard of the HPV vaccination programme	4	1.25
At school	193	60.12
Clinic	63	19.63
Pamphlet	12	3.74
Radio or Television	49	15.26

HPV, human papillomavirus.

### Awareness of human papillomavirus and the human papillomavirus vaccination

The results of the awareness of parents and caregivers on HPV and the HPV vaccination programme are depicted in Table 3. These findings show that 64% (*n* = 206) of parents knew that HPV causes cancer of the cervix and that there is a vaccine to prevent cervical cancer 67% (*n* = 216). Sixty per cent heard about the HPV vaccination programme from the schools (see Table 2), and overall, at least 86% (*n* = 275) heard about the programme. Furthermore, 71% (*n* = 227) were aware that the programme is also available in public health care facilities. A total of 80% (*n* = 258) knew that HPV vaccination is offered free of charge and that they ought to give permission for their daughters to be vaccinated (93%, *n* = 297).

**TABLE 3:** Awareness of human papillomavirus and the human papillomavirus vaccination among parents and caregivers of girls eligible for receiving human papillomavirus vaccination in Tshwane.

Statements	Yes		No	
	*n*	%	*n*	%
1. I know about a virus that causes cancer of the cervix in women (HPV).	206	64	115	36
2. There is a vaccine that prevents cancer of the womb or cervix in women.	216	67	105	33
3. I have heard of the school vaccination programme against HPV.	275	86	46	14
4. The HPV vaccination programme is available in public health care facilities.	227	71	94	29
5. There is a need to pay a minimal fee for my daughter to be vaccinated in the public vaccination programme.	63	20	258	80
6. I must give permission for my daughter to be vaccinated.	297	93	24	7
7. My daughter must be 9 years and older to be vaccinated.	279	87	42	13
8. My daughter only needs one injection of the HPV vaccine, then she will be protected against HPV.	184	57	137	43

HPV, human papillomavirus.

Also, 87% (*n* = 279) of the parents knew that their daughters had to be 9 years and older to be vaccinated. Only 43% (*n* = 137) knew that their daughter requires two doses of the HPV vaccine to be protected against HPV. Most parents understood that they were required to allow their daughters to be vaccinated during the HPV vaccination programme.

Data regarding the knowledge of parents and caregivers on HPV and the HPV vaccination programme being held in schools are shown in Table 4.

**TABLE 4:** Knowledge of parents and caregivers on human papillomavirus and the human papillomavirus vaccination programme.

Statements	Correct		Incorrect	
	*n*	%	*n*	%
1. Why are young girls vaccinated with the HPV vaccine?	292	91	29	9
2. How many times does the HPV vaccination programme take place in schools in a year?	95	30	226	70
3. How many injections or doses are needed for your daughter to be fully immunised against HPV in South Africa?	85	26	236	74
4. Who should make the decision on vaccination?	312	97	9	3
5. I have enough information about the HPV vaccine to decide whether to give it to my daughter or not.	215	67	106	33
6. The decision to vaccinate my daughter, who is a minor and relies on me as her parent or caregiver, rests solely with me.	294	92	27	8

HPV, human papillomavirus.

Although 70% (*n* = 226) reported not knowing the frequency of school-based vaccination, and 74% (*n* = 236) were unaware of the required number of doses for complete HPV immunisation, the majority 97% (*n* = 312) recognised that the decision to vaccinate their children ultimately rests with them as parents. Ninety-seven per cent of girls knew that parents are the ones to decide whether their children are to be vaccinated or not. Two-thirds of the parents 67% (*n* = 215) indicated that they had enough information to decide whether to vaccinate their daughters or not. However, the majority of them 92% (*n* = 294) knew that the final decision for vaccination of their daughters lies with them as parents.

### Acceptability of the human papillomavirus vaccination programme in schools

Table 5 shows the level of acceptability of the HPV vaccination among parents of girls eligible to receive the HPV vaccination. Most parents felt that the policy of providing the HPV vaccination to every girl aged 9 years and above is a good initiative (88%, *n* = 281); it is in the best interest of their daughters to receive the vaccine (89%, *n* = 287). Furthermore, 86% (*n* = 275) parents and caregivers thought that the HPV vaccine would benefit their daughters in future, and 76% (*n* = 243) believed that the vaccine is safe and tested before administration to girls in schools. Sixty-four per cent (64%) of the parents believed that the vaccine does not influence their daughters’ sexuality or any other behaviour relating to sexual orientation.

**TABLE 5:** Acceptability of human papillomavirus vaccination among parents and caregivers of girls eligible to receive the human papillomavirus vaccine in Tshwane (*N* = 321).

Variables	Strongly disagree (1)		Disagree (2)		Neutral (3)		Agree (4)		Strongly agree (5)		Mean score	
	*n*	%	*n*	%	*n*	%	*n*	%	*n*	%	Mean	s.d.
1. The policy of giving the HPV vaccination to every girl who is 9 years and older is a good initiative.	4	1.25	7	2.18	29	9.03	157	48.91	124	38.63	4.21	0.79
2. It is in the best interest of my daughter to receive the HPV vaccination.	3	0.93	8	2.49	23	7.17	157	48.91	130	40.5	4.26	0.77
3. This vaccine will benefit my daughter in the future as it is given as a preventative measure.	3	0.93	8	2.49	35	10.9	136	42.37	139	43.3	4.25	0.82
4. The vaccine is safe and tested before it was approved to be administered to girls in schools.	6	1.87	3	0.93	69	21.5	139	43.3	104	32.4	4.03	0.86
5. The vaccine has no influence on my daughter’s sexuality or any other sexual orientation behaviour.	11	3.43	25	7.79	81	25.23	109	33.96	95	29.6	3.79	1.06
6. If the government offers free HPV vaccination, I will vaccinate my daughter.	4	1.25	14	4.36	21	6.54	151	47.04	131	40.81	4.22	0.85
7. I would pay for my daughter to be vaccinated if she does not qualify to receive the vaccine for free.	24	7.48	73	22.74	62	19.31	95	29.6	67	20.87	3.34	1.24
8. I will allow my daughter to be vaccinated during the HPV vaccination programme.	9	2.8	22	6.85	75	23.36	142	44.24	73	22.74	3.77	0.97
9. I will recommend other parents to allow their daughters to be vaccinated during the HPV vaccination programme.	2	0.62	5	1.56	20	6.23	163	50.78	131	40.81	4.3	0.71
10. Every child who is 9 years and older should be vaccinated with the HPV vaccination in schools.	8	2.49	7	2.18	38	11.84	159	49.53	109	33.96	4.1	0.87
**Overall acceptability score**	**-**	**-**	**-**	**-**	**-**	**-**	**-**	**-**	**-**	**-**	**4.03**	**0.89**

HPV, human papillomavirus; s.d., standard deviation.

According to most parents, they would vaccinate their children even if it is free (88%, *n* = 282); however, about 50% (*n* = 162) indicated they would pay for their daughters to be vaccinated if they did not qualify to receive free vaccines. Most parents (88%, *n* = 284) indicated that they would allow their daughters to be vaccinated, while a much higher (87%, *n* = 278) reported they would recommend the vaccine to other parents. Lastly, 83% of parents felt that every child who is 9 years and older should be vaccinated against HPV at schools. The overall acceptability score of 4.03 (0.89) generally suggests that parents accept the HPV vaccination programme in schools.

**FIGURE 1:**
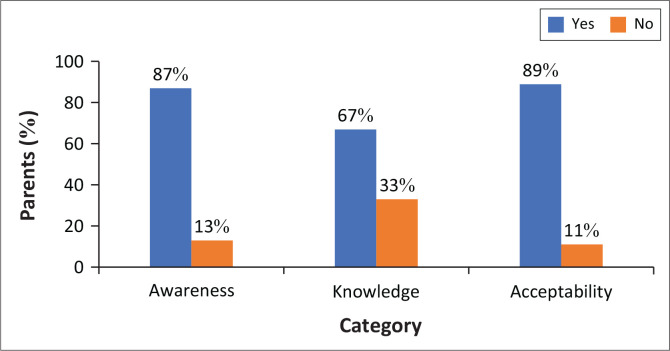
Overall level of awareness, knowledge and acceptability of human papillomavirus vaccination.

Overall level of awareness, knowledge and acceptability of the HPV vaccination programme in the schools is shown in Figure 1.

The overall results of awareness, knowledge and acceptability of the HPV vaccination programme depicted in Figure 1 show that eighty-seven per cent (87%, *n* = 279) of parents were aware of the vaccination programme in schools, 67% (*n* = 215) had sufficient knowledge to decide on vaccinating their daughters or not, whereas 89% (*n* = 286) were willing to accept implementation of the vaccination programme in schools. Overall, 11% of parents or caregivers seem hesitant and sceptical about the HPV vaccination.

### Parental factors associated with the acceptability of the HPV vaccination programme

Factors associated with the acceptability of HPV vaccination among parents and caregivers are shown in Table 6. The univariate analysis at a relaxed *p*-value of 0.25 shows that the acceptability of the HPV vaccine is likely to be significantly associated with race (being black) and source of HPV information (school, radio or television). This shows a promise of being significant, and they were subsequently included in the multivariable analysis. The awareness and knowledge of HPV and the HPV vaccination programme were also found to be significantly associated with the acceptability of HPV vaccination.

**TABLE 6:** Parental factors associated with the acceptability of the HPV vaccination programme.

Variables	Acceptability of HPV vaccination				Univariate logistic regression			Multivariable logistic regression		
	Yes		No		uOR	95% CI	*p*-value	aOR	95% CI	*p*-value
	*n*	%	*n*	%						
**Gender**										
Male	18	6	2	6	Ref	-	-	Ref	-	-
Female	269	94	32	94	0.93	0.21; 4.21	0.929	0.42	0.72; 2.41	0.329
**Race**										
White parents or caregivers	12	4	3	9	Ref	-	-	Ref	-	-
Parents or caregivers of mixed race	16	6	3	9	1.331	0.28; 7.80	0.750	5.56	0.56; 55.29	0.143
Black parents or caregivers	259	90	28	82	2.31	0.62; 8.69	0.215*	6.45	1.10; 37.76	0.039**
**Marital status**										
Single	194	68	24	71	Ref	-	-	Ref	-	-
Married	82	29	10	29	1.01	0.46; 2.22	0.971	1.99	0.68; 5.80	0.209
Divorced	7	2	0	0	-	-	-	-	-	-
Widowed	4	1	0	0	-	-	-	-	-	-
**Where did you find out about the HPV vaccination programme? (HPV information source)**										
Never heard of the HPV programme	2	1	2	6	Ref	-	-	Ref	-	-
At school	175	61	18	53	9.72	1.29; 73.22	0.027*	1.72	0.14; 20.94	0.671
Clinic	55	19	8	23	6.88	0.85; 55.89	0.071*	0.83	0.06; 11.39	0.891
Pamphlet	10	3	2	6	5	0.42; 59.66	0.203*	2.68	0.12; 58.32	0.530
Radio or Television	45	16	4	12	11.25	1.23; 102.62	0.032*	1.65	0.11; 24.28	0.715
**HPV awareness**										
Not aware	24	8	19	56	Ref	-	-	Ref	-	-
Aware	263	92	15	44	13.88	6.26; 30.76	0.000*	7.17	2.67; 19.07	0.000**
**HPV knowledge**										
Not knowledgeable	78	27	29	85	Ref	-	-	Ref	-	-
Knowledgeable	209	73	5	15	15.54	5.80; 41.58	0.000*	10.98	3.60; 33.49	0.000**

HPV, human papillomavirus; uOR, unadjusted odds ratio; aOR, adjusted odds ratio; CI, confidence interval.

* significant at *p* < 0.25;

** significant at *p* < 0.05.

At the multivariable level, race remained a significant predictor with black parents being 6 times more likely (adjusted odds ratio [aOR] = 6.45; *p* = 0.039; 95% confidence interval [CI]: 1.10–37.76) to accept the HPV vaccination programme than their white and coloured counterparts. Additionally, parents who were aware of the HPV vaccination programme were seven times more likely to accept the HPV vaccination than those who were not aware (aOR = 7.17; *p* < 0.000; 95% CI: 2.67–19.07). Lastly, having HPV knowledge was also a significant predictor of acceptability of the programme (aOR = 10.98; *p* < 0.000; 95% CI: 3.60–33.49). The results of the Hosmer–Lemeshow goodness–of–fit test, with a *p*-value of 0.0520, show that the model adequately fits the data, and the sample size is reasonably large.

## Discussion

This study assessed awareness, acceptability and factors influencing the acceptability of the HPV vaccination programme among parents of Grade 5 girls attending public schools in Tshwane, Sub-district 6. The findings revealed high awareness levels, with 87% of parents aware of the HPV vaccination programme. Additionally, 86% had heard about the programme through schools, 64% knew that HPV causes cervical cancer, and 67% recognised the vaccine as a preventive measure.

### Awareness and source of information of the human papillomavirus vaccination programme

These results align with studies conducted in other regions. For instance, in Kenya, Mwenda et al. (2023) found 88% awareness among educators (who were also parents), with 92% acknowledging the vaccine’s role in cervical cancer prevention. However, contrasting findings were observed in a South African study by Tshivhase et al. (2023), where only 28.6% of participants had heard of HPV, and fewer than 5% understood its association with cervical cancer. Similarly, another study conducted in KwaZulu-Natal, South Africa, has shown that 31% of urban and 26% of rural residents were aware of HPV, while 72% were unaware of school-based vaccination programmes for girls aged 9 and older.

Globally, low awareness persists: studies in Malaysia (Wong, Wong & Mohamad 2021) and China (Li, Zhang & Wang 2022) documented HPV familiarity rates of only 18% – 24% among caregivers with vaccine awareness at 22% – 25%. These disparities highlight potential information gaps, increasing the risk of HPV exposure and missed opportunities among the uninformed population.

The higher awareness observed in this study may be attributed to targeted HPV campaigns by school health teams and health promoters. These initiatives included parent meetings, radio/television announcements, pamphlets and health facility engagements. Notably, participants who received information *via* mass media (radio/television) were 1.8 times more likely to accept vaccination than those informed through other channels (Ndwandwe et al. 2023), reinforcing mass media’s critical role in health promotion.

### Parental knowledge and decision-making on the human papillomavirus vaccination

This study found that 67% of parents believed they had sufficient information about HPV and the programme to make an informed decision regarding their daughters’ vaccination. These findings align with Milondzo et al. (2023) in South Africa, where 75% of parents demonstrated good knowledge of HPV and its vaccination. However, contrasting results were reported by Tshivhase, Makhado and Mabunda (2022), where 89% of participants lacked adequate knowledge, leading to 85% hesitancy towards vaccine acceptance.

Despite overall awareness, gaps in detailed knowledge persisted. Only 43% of parents knew that a single dose is insufficient for full protection, and just 26% were aware of the required number of doses. This suggests that while general awareness is improving, specific knowledge about the vaccination regimen remains lacking, as also observed in recent studies (Ndwandwe et al. 2023; Wiyeh et al. 2023).

Nevertheless, Kisaakye et al. (2022) noted that even with limited knowledge, many parents expressed interest in learning more and were willing to vaccinate their children. Given that parents are critical stakeholders in vaccination decisions, ensuring they receive accurate, culturally tailored information is essential (Jensen et al. 2022; Okunade et al. 2023). As highlighted by Ndou et al. (2021), acceptability hinges on understanding – the disease, vaccine benefits and dosing regimen.

### Factors associated with the acceptability of the human papillomavirus vaccination programme

This study found high levels of HPV vaccine acceptability with 89% of parents approving vaccination. Most parents were willing to have their daughters vaccinated in schools and recommend vaccination to others. These findings are consistent with the results from sub-Saharan Africa, where acceptance rates of 70% – 92% have been documented (Kisaakye et al. 2022; Ndwandwe et al. 2023). In contrast, significantly lower acceptance (28% – 35%) persists in some Asian populations (Li et al. 2022; Wong et al. 2023).

This study indicates that parents with knowledge about HPV and the HPV vaccination programme were likely to accept it. This aligns with Jensen et al. (2022), who found that mothers with poor knowledge were 2.3 times less likely to accept the vaccination. Similarly, Yusuf et al. (2024) reported that higher education levels reduced vaccine hesitancy. Interestingly, Wiyeh et al. (2023) demonstrated that brief educational interventions increased HPV acceptance by 40% in South Africa. While Gray and Fisher (2023) found parental knowledge was not significant in multivariable analysis (aOR = 0.94, *p* = 0.48), the strong association in the current study likely reflects the effectiveness of school-based outreach, where healthcare workers actively engage parents (Ndou et al. 2021; Okunade et al. 2023). Racial disparity in the HPV vaccination acceptance was observed in this study. Black parents were more likely to accept the HPV vaccination for their daughters than the other racial groups. This contrasts with Sonawane et al. (2023) and Sokale, Moreno and O’Leary (2024), who found lower uptake among black parents in the US. However, Tshivhase et al. (2022) reported similar findings to the current study in South Africa, attributing higher acceptance to trust in healthcare systems and community-based messaging.

### Implications and recommendations

#### Implications

**High awareness but knowledge gaps**

- While 87% of parents were aware of the HPV vaccination programme, only 43% knew that two doses were required for full protection. This indicates that awareness campaigns have been successful in reaching parents, but detailed knowledge about dosage and scheduling remains insufficient.
- Misinformation or a lack of clarity may lead to incomplete vaccination compliance, reducing the vaccine’s effectiveness.

**Strong acceptability but racial disparities**

- Black parents were six times more likely to accept the HPV vaccination than other racial groups, suggesting cultural or community-based trust in public health initiatives.
- However, white and mixed-race parents showed lower acceptance, indicating a need for targeted messaging to address hesitancy in these groups.

**School-based outreach is effective**

- About 60% of parents heard about the HPV vaccination through schools, reinforcing that school health programmes are a key channel for disseminating HPV-related information.
- Mass media (radio or TV) also played a role, but school engagement remains the most impactful.

**Employment and education influence decision-making**

- A majority of participants were unemployed (55%), and 28% had only a high school education, which may affect their ability to access or interpret health information.
- Lower-income and less-educated parents may rely more on school-based programmes, whereas higher-income groups might seek private healthcare alternatives.

**Religious and sociocultural factors**

- Ninety-four per cent of participants were Christian, and only a small minority expressed concerns about the HPV vaccination influencing sexual behaviour. However, in other studies, religious beliefs have been linked to vaccine hesitancy, suggesting that faith-based engagement strategies could further improve acceptance.

#### Recommendations

**Enhance parental education on the human papillomavirus vaccination**

- School-based workshops and pamphlets should clearly explain the two-dose requirement and the importance of completing the vaccination schedule.
- Use simple, visual aids (e.g. infographics) to improve understanding among parents with lower education levels.

**Strengthen community and mass media campaigns**

- Radio and TV messages should be expanded to reinforce school-based information.
- Engage community leaders and religious figures to advocate for HPV vaccination, addressing cultural or faith-based concerns.

**Targeted messaging for hesitant groups**

- Tailored communication strategies should be developed for white and mixed-race parents, addressing their specific concerns (e.g. vaccine safety, perceived necessity).
- Healthcare providers should engage in one-on-one discussions with hesitant parents to dispel myths.

### Strengths and limitations of the study

This study enhances the understanding of key factors influencing the acceptability of the HPV vaccination programme. The findings offer valuable insights to strengthen vaccination campaigns and improve coverage rates. However, the study has limitations inherent to its cross-sectional design, which precludes longitudinal assessment of vaccination acceptance over time. While this study ensured representativeness by stratifying schools into quintiles, participation was voluntary, limited to parents of eligible daughters who were willing to enrol. This self-selection process may introduce volunteer bias, potentially affecting the generalisability of the results.

## Conclusion

Despite the high level of awareness and acceptability of the HPV vaccination among parents in Tshwane Metropolitan, knowledge gaps and racial disparities persist. The study underscores that informed parents are more likely to accept the school-based vaccination programme. Furthermore, this study emphasises the need to enhance school-based educational campaigns using multi-platform strategies such as parents’ meetings, mass media outreach and pamphlet distribution in the community, among other things. Empowering parents with comprehensive information and addressing hesitancy in specific groups can enhance informed decision-making and further improve HPV vaccine uptake. Such efforts would not only address knowledge disparities but also bolster trust and increase participation, ensuring the long-term success of the school-based HPV vaccination programme. Policymakers and healthcare providers should prioritise these strategies to ensure broader protection against cervical cancer.
